# Risk stratified breast cancer screening: UK healthcare policy decision-making stakeholders’ views on a low-risk breast screening pathway

**DOI:** 10.1186/s12885-020-07158-9

**Published:** 2020-07-22

**Authors:** Lorna McWilliams, Victoria G. Woof, Louise S. Donnelly, Anthony Howell, D. Gareth Evans, David P. French

**Affiliations:** 1 Manchester Centre for Health Psychology, Division of Psychology and Mental Health, School of Health Sciences, Faculty of Biology, Medicine and Health, University of Manchester, MAHSC, Oxford Road, Manchester, M13 9PL UK; 2 NIHR Manchester Biomedical Research Centre, Manchester Academic Health Science Centre, Manchester University Hospitals NHS Foundation Trust, Manchester, England; 3 Nightingale & Prevent Breast Cancer Research Unit, Manchester University NHS Foundation Trust, Southmoor Road, Wythenshawe, Manchester, M23 9LT UK; 4 NIHR Greater Manchester Patient Safety Translational Research Centre, Centre for Mental Health and Safety, School of Health Sciences, Faculty of Biology, Medicine and Health, University of Manchester, MAHSC, Oxford Road, Manchester, M13 9PL UK; 5 Department of Genomic Medicine, Division of Evolution and Genomic Science, Manchester Academic Health Science Centre, University of Manchester, Manchester University NHS Foundation Trust, Oxford Road, Manchester, M13 9WL UK

**Keywords:** Risk stratification, Breast cancer, Screening, Implementation, Risk assessment

## Abstract

**Background:**

There is international interest in risk-stratification of breast screening programmes to allow women at higher risk to benefit from more frequent screening and chemoprevention. Risk-stratification also identifies women at low-risk who could be screened less frequently, as the harms of breast screening may outweigh benefits for this group. The present research aimed to elicit the views of national healthcare policy decision-makers regarding implementation of less frequent screening intervals for women at low-risk.

**Methods:**

Seventeen professionals were purposively recruited to ensure relevant professional group representation directly or indirectly associated with the UK National Screening Committee and National Institute for Health and Care Excellence (NICE) clinical guidelines. Interviews were analysed using thematic analysis.

**Results:**

Three themes are reported: (1) producing the evidence defining low-risk, describing requirements preceding implementation; (2) the impact of risk stratification on women is complicated, focusing on gaining acceptability from women; and (3) practically implementing a low-risk pathway, where feasibility questions are highlighted.

**Conclusions:**

Overall, national healthcare policy decision-makers appear to believe that risk-stratified breast screening is acceptable, in principle. It will however be essential to address key obstacles prior to implementation in national programmes.

## Background

The National Health Service Breast Screening Programme (NHSBSP) currently invites women aged 50–70 years registered with a general practitioner (GP) in the United Kingdom to attend for 3-yearly mammograms. The interval, using the best available evidence [1, 2], was based on the Forrest Report [3]. Most other countries invite women to screening bi-annually due to later interval cancer data; however, a subsequent UK trial demonstrated 3 years to be acceptable due to the relatively small effect of more frequent intervals on breast cancer mortality [4]. Since, there has been considerable debate around the harms and benefits of breast screening [5]. This has primarily centered on overdiagnosis i.e. women who receive treatment for malignancies that would have never presented symptomatically without screening [6], and false positive test results [7].

One way to improve this balance is to risk-stratify breast screening. It is recommended that women at high-risk of breast cancer are offered more frequent screening or chemoprevention [8]. By contrast, women at low-risk of developing breast cancer could experience greater harms, as tumours they develop are much more likely to be early stage and slow-growing [9]. Consequently, low-risk women might benefit from attending screening less frequently. However, there is no systematic process for identifying either group in routine screening.

Recent evidence has demonstrated the predictive value of breast cancer risk models, such as Tyrer-Cuzick (TC), that provide women with individual risk estimates [10–12]. The models, based on known risk factors, include mammographic density, hormonal and reproductive information (for example, age of menarche), genetic information and family history. A recent study within the UK NHSBSP found that 13.5% received a 10-year breast cancer risk estimate of less than 1.5% using TC including mammographic density [13]. This low-risk threshold is equivalent to the mean risk of women aged 40-years who are not yet offered routine breast screening in the NHSBSP [14]. Reducing screening for such a proportion of women could result in substantial savings. Existing data suggest that risk-stratification is potentially cost-effective [15, 16], although reducing the screening frequency for low-risk women could potentially improve this further.

Despite these potential benefits, limited research has explored the acceptability of risk-stratified breast screening. Research to date has focused on genomics-based risk stratification or identifying prevention strategies for high risk groups [17–21]. There appears to be no published evidence using in-depth methods to elicit the views of professionals involved in national screening programme decision-making, healthcare policy and implementation with a focus on low-risk. Understanding the views of such individuals will allow progress to be made on whether and how to implement low-risk pathways, and identify evidence gaps. This is timely given that the UK healthcare setting is more advanced in assessing the feasibility of risk stratification with current trials assessing the implementation of risk-stratified breast screening [22, 23]. This study aimed to describe the perspectives of individuals who advise and make healthcare policy decisions including breast cancer screening.

## Methods

### Design, setting and participants

A cross-sectional, qualitative design using semi-structured interviews was used. To gain diversity of views across relevant professions, purposive sampling using two criteria was used to recruit UK-based healthcare policy advisors and decision-makers related to cancer screening and clinical guidelines. All individuals were identified through publically available current or recent membership/guideline author lists relevant to national cancer screening programmes. Lists were obtained for the UK National Screening Committee (UKNSC), UKNSC Adult Reference Group, Advisory Committee on Breast Cancer Screening as well as the National Institute for Health and Care Excellence (NICE) Committees and Guideline writing groups. Secondly, we aimed to produce a sample that was diverse in terms of professional/disciplinary background i.e. from the above pool of people involved in breast cancer and/or breast cancer screening. Sampling aimed also to ensure diversity in relation to expertise in screening programme management, public health, radiology, radiography, nursing, surgery, health economics, epidemiology, statistics, medical ethics and primary care. Potential participants were invited to participate by email (LM, VGW) and up to three reminder emails were sent approximately 2 weeks apart. The email template was signed by another co-author (DGE) who has a national profile in cancer screening.

### Procedure

An interview topic guide (see Additional File 1) was developed to guide all interviews based on relevant literature and considering implementing change to healthcare services. This was piloted with two breast cancer prevention oncologists to assess wording of questions, prompts and flow before finalising. Questions related to: feasibility, implementation, low-risk threshold, screening interval length, information provision, informed decision-making and potential implications of stratifying screening for low-risk women (from service, policy and public perspectives). The guide was used flexibly to ensure all topics of interest plus any new points raised by participants were explored in each interview. Interviews were conducted face-to-face in participants’ workplace, home or by telephone. The study received ethical approval and all participants provided informed consent prior to interviews. Interviews were audio-recorded and conducted by two female researchers (LM, VGW) with post-graduate qualitative research (psychology discipline) training. After each interview, LM and VGW took detailed notes and held debrief discussions. Interviews continued until the research team agreed (LM, VGW, DPF) that data sufficiency had been achieved, whereby adequate data had been collected and from a diverse sample of professions [24]. This was determined by discussing the overlap or discrepancies between cases, i.e. nothing new being introduced during interviews, until it appeared no further interviews were required. For example, additional perspectives from health economics and epidemiology were sought towards the end of data collection before recruitment was finalised. All interviews were transcribed by an external transcription agency.

### Analysis

Data were analysed using six stages of thematic analysis from an essentialist perspective, which aims to identify patterns across the dataset allowing researchers to report the experiences and realities from participants as they appear in the data [25]. This manifest-level analysis was open-ended (inductive) rather than using an existing frame to code the data. Trancripts were checked for accuracy by listening to all interviews (LM, VGW). Transcripts were then initially read multiple times (LM) to gain familiarity and coded using Nvivo-11 software. Three transcripts were double coded descriptively (LM, VGW) to discuss the emerging coding framework; discrepancies were resolved and agreed upon before being applied to the remaining transcripts. The coding framework was discussed with study team members throughout analysis in face to face meetings following analysis documents shared in advance over email (LM, VGW, LSD, DPF) and used to generate themes. The thematic structure was compared across transcripts to account for similarities and distinctions between participants. Any differences of views were discussed and themes refined in light of discussion before being finalized and agreed upon by the entire study team. An abstract including the findings was sent to all participants to invite them to opt to be named in acknowledgements of publications. The themes best describe the participant’s views on implementing an extended breast screening interval for low-risk women.

## Results

### Sample

In total, thirty people were invited and 17 people took part (females = 11). Five people did not respond and eight people declined. Reasons provided for non-participation were related to conflict of interest (3 cases) or lack of time. Interviews lasted 27 to 124 min; seven were conducted by telephone. A wide variety of professions associated with breast cancer screening were represented. Specific roles included six breast cancer healthcare professionals within radiology, oncology, radiography, nursing and surgery; six senior academics: ethics, epidemiology, statistics and health economics; and five breast screening programme operations/management professions including user involvement. All participants were involved directly or indirectly in the UKNSC, NICE, UKNSC Adult Reference Group or Advisory Committee on Breast Cancer Screening.

Overall, participants found it difficult to discuss implementing a low-risk pathway from wider considerations about risk-stratified screening. However, participants considered specific aspects of how low-risk is defined and might be implemented at population-level. Three themes with nine sub-themes are presented (see Table 1). Quotes are identified by participant profession type and participant number.

**Table 1:** Thematic Structure

Theme	Sub-theme
1. Producing the evidence defining low risk	1.1. Overcoming reservations about evidence accuracy
	1.2 Determining a risk threshold and interval length
	1.3 Risk stratification should be cost-effective
2. The impact of risk stratification on women is complicated	2.1 Managing women as individuals
	2.2 Balancing the harms and benefits
	2.3 The ability to make autonomous decisions
3. Practically implementing a low-risk pathway	3.1 Initial feasibility work required
	3.2 Communication is essential
	3.3 Considering service implications

### Theme 1: producing the evidence defining low-risk

Participants discussed concerns about the strength of evidence available to successfully implement risk-stratified screening including extending screening intervals for women at low-risk of breast cancer.

#### Sub-theme 1.1: overcoming reservations about evidence accuracy

Although there was broad recognition that risk-stratification could be applied to future breast screening programmes, all participants were unconvinced there is evidence that suggests a less frequent screening interval for low-risk women is currently acceptable outwith a research context. Their key concern focused on the possibility, using existing risk models and with limited knowledge around the natural history of the disease, of identifying a risk low enough that women would not have greater chance of being diagnosed with higher grade, aggressive breast cancers compared to other risk groups.

*I’m not aware that it’s possible to say that because you’re a low-risk woman, if you do get a cancer, it’s going to be that kind of cancer and not this kind of cancer* (Healthcare professional; 2035).

Participants therefore expressed that demonstrating accuracy of whichever risk model is applied during implementation will provide crucial evidence to identify a ‘true’ low-risk cohort. Some participants reported that this should then inform a trial assessing the harms and benefits of extended intervals. However, difficulties applying gold standard trial designs (i.e. randomised controlled trials) in screening and using mortality reduction as the primary outcome was made evident given the length of follow-up required to determine effectiveness.

#### Sub-theme 1.2: determining a risk threshold and interval length

Despite the acknowledgement that a group of women within any given population would have lower risk, participants found it difficult to suggest a threshold and interval length to define a safe low-risk pathway. However, participants described ways in which these (beyond current national programme intervals) could be established. Many participants reported the expectation to see data demonstrating a minimal impact of extending the screening interval for low-risk women on subsequent interval cancer rates. This was viewed by most as a proxy measure for mortality evidence although some participants did not feel they had sufficient subject knowledge to comment in detail.

*… it will be important to convey that it’s not just about the pick-up rate of numbers of diagnoses but it’s about the severity of those diagnoses and so if the modelling captures the fact that actually the reduction in pick-up is not translating into missing aggressive or poor prognosis cancers, and that there’s no impact on the mortality, then I guess that’s the important thing …* (Healthcare professional; 2032).

Several participants reported age at risk assessment being an important consideration over and above the risk model used given that mammography is less sensitive in younger women and they are more likely to develop aggressive tumours.

*… age but not just age in terms of the effect of age on your risk. Age in terms of its effect on the likely progression rate of cancer … someone that’s 50, if they had very low-risk, I would feel a bit more worried about extending the interval beyond 3 years just because […*] *the proportion of hormone dependent cancers increases with age. The younger you are, the more likely it is to be non-hormone dependent, faster moving*. (Academic; 2022).

Some participants stressed the importance of modelling the proportion of women per risk group to determine whether any had a greater chance of subsequent interval cancers. All risk groups were often considered rather than low-risk in isolation given that breast screening is population-level.

*… you should equalise interval cancers and I would like to see what different screening intervals you would get if you worked on equalising interval cancers.* (Academic; 2034).

Tensions surrounding the lack of evidence led to speculation about whether the harms of attending breast screening may outweigh benefits for low-risk women. Given that mortality reduction is the ultimate purpose of breast screening, several participants expressed the desire to know the impact of extended screening intervals on this.

*… if you were going to extend the interval a very real question is, well, is it worth bothering at all and you can’t assume that it is, it might be they’re better off not going at all, because if it doesn’t reduce mortality and they get a dose of radiation […*] *then all you get are the disadvantages of screening without the advantages*. (Screening operations/ management; 2026).

There was no consensus about what interval length could be used. Even though some participants acknowledged other screening programmes (such as cervical) have varied screening frequencies, current breast screening intervals are long established underpinning reluctance of a substantial departure. Some participants found it difficult to comment at all on a length longer than three-years due to lack of safety confirmation whilst one participant felt it should link with current programme delivery to minimize disruption.

*… already the UK programme gets criticised for having three yearly intervals because most European programmes have a two-year interval and they feel that 3 years, there’s much less of a safety net. You know, if a cancer’s missed at one screen there’s still quite a good chance that it’ll be still at an early stage at the next one two years later. But if the next one’s three years later there’s a bit more concern. So, I would think there’s not that much point going beyond 4 years.* (Academic; 2022).

#### Sub-theme 1.3: risk stratification should be cost-effective

Participants explained that cost-effectiveness evidence is possibly more important for publicly funded healthcare systems and almost all participants elaborated that this is vital when deciding whether to implement at policy level.

*… it depends on the healthcare system, because there will be very different motivators to things like having diagnostic tests and more frequent screening when you’re having to pay for it […*] *when it’s free it’s got to be cost effective and it’s got to be evidence based.* (Screening operations/management; 2027).

Cost-effectiveness driving the decision to increase screening intervals for low-risk women was however viewed with caution regarding how this may be perceived by stakeholders. Cost-effectiveness modelling was therefore considered by some as a step that should take place after it has been shown that risk stratification is accurate and clear communication strategies are in place.

*… people might be concerned that the reason this was being done, was to save money, and not necessarily for a health benefit for the wider population, or particularly of benefit for the woman of low-risk.* (Academic; 2024).

### Theme 2: the impact of risk stratification on women is complicated

Participants discussed the ways in which stratified breast screening for low-risk women could be received by those invited and wanted to know how acceptable this would be to women themselves.

#### Sub-theme 2.1: managing women as individuals

The ‘personal’ aspect of risk stratification was viewed as a positive step for breast screening by acknowledging that not all women are the same, as with other disease pathways. However, many participants acknowledged that women will likely discuss risk stratification with each other. This may lead to confusion given the number of potential risk-based pathways.

*...is it right to impose the same screening option across all different variations of women, when their backgrounds are different? Personally, I don’t think it is, I think we have come to a time where we could make things more personalised to that individual woman’s needs.* (Healthcare professional; 2030).

Participants identified individual beliefs about risk and knowledge of breast cancer and screening as key factors that will impact how women could respond to low-risk stratification. Participants felt this should guide the development of communication and information about a low-risk pathway to facilitate understanding. A personal approach about being low-risk was considered most appropriate to communicate with women, particularly for groups who may already be disengaged with screening.

*… there’s a strong perception of susceptibility to breast cancer in the population […*] *people still know people who have died horrible deaths from breast cancer, and so it’s a high sort of perceived severity as well.* (Healthcare professional; 2035).

#### Sub-theme 2.2: balancing the harms and benefits

The potential harms and benefits of extended screening intervals for low-risk women were identified and discussed by all. Although some women could feel reassured that they have a lower risk of breast cancer leading to reduced worry, women may equally feel afraid about having reduced screening.

*… there are two ways of looking at it, aren’t there, from a woman’s point of view. She can either look at it to say, ‘yippee! I’m such low-risk; I don’t need another screening for five years.’ Or she could look at it to say, ‘oh that’s a bit worrying, I thought screening was only any good up you know, at three yearly intervals. And here I am being put on to five years.’* (Healthcare professional; 2025).

All participants described positive aspects of low-risk screening where women would have less inconvenience of attending, fewer occasions undergoing mammogram discomfort, reduced chance of additional tests and overdiagnosis. Yet the risk that women may still experience an interval cancer diagnosis was always acknowledged and one participant felt that low-risk women would still be at risk of overdiagnosis, but just a delayed diagnosis. It was felt by some that this could lead to negative psychological consequences for women and, question their confidence in breast screening overall.

*… the obvious harm is a woman’s breast cancer that could’ve been screen detected isn’t. You don’t know that’s harmful because you don’t know what type of cancer it is; […*] *there’d be psychological harm to that as well as ‘I signed up to having this less frequent mammograms and I possibly could’ve had my breast cancer detected two years earlier’.* (Healthcare professional; 2029).

Participants voiced concerns that women at low-risk may be overly reassured and misunderstand that although below average; they still have *some* breast cancer risk. In particular, many expressed a low-risk pathway should include breast awareness education and resources about changes in risk to mitigate this.

*… some people might think, ‘oh, I’m low-risk, I don’t need to go at all’, and then might not respond when you actually send the 5 year or longer letter through.* (Screening operations/management; 2033).

#### Sub-theme 2.3: the ability to make autonomous decisions

All participants considered individual versus population-level consequences of risk-stratified screening. There was a sense of conflict when considering what is ethically right versus a feasible pathway to implement. Participants explained that women should have informed choice about having their risk assessed and ability to weigh up pros and cons of less frequent screening yet, it was acknowledged by some that having choice to remain on current screening intervals if low-risk could make risk stratification untenable.

*… you need to go and check with people, enough, I would say, to say ‘Does she understand and then can she make an informed choice?’* (Screening operations/management; 2021).

To add further complexity to decision-making, women were often categorised into two groups, those not yet invited for screening and those already ‘in’ screening. Although introducing a low-risk pathway to women first invited to screening was viewed favourably, some participants expressed the difficulties of managing screening being ‘taken away’ from women already in the programme. Other participants were concerned that even if it were more feasible to introduce screening only to those entering the programme, this would create inequity of access given that all women would not have the opportunity of risk assessment. There was no real consensus on how best to introduce a low-risk pathway aside from stressing the importance of obtaining the views of women themselves.

***…****do you start the new regime for just new women coming into the programme and continue the current policy for those existing in the screening programme? If you do that you create an inbuilt inequality and a two-tiered service. Or do you allow women the choice to be given a baseline test and then a new regime, or allow them to continue on their old one?* (Screening operations/management; 2027).

### Theme 3: practically implementing a low-risk pathway

All participants considered implementing risk-stratified screening for low-risk women and the challenges this would entail.

#### Sub-theme 3.1: initial feasibility work required

Participants considered what a low-risk pathway could look like practically within breast screening, highlighting key issues concerning infrastructure capability and the need for careful organisational preparation. All participants acknowledged that adapting a relatively straightforward, mostly universal programme to one involving multiple pathways would not be easy and discussed this with pessimism. Feasibility work and pilot testing were viewed as fundamental to minimise issues. One participant highlighted that even if pilots are successful, continued implementation evaluation would be worthwhile during wider rollouts.

*… you can’t change a direction of a cruise ship overnight, you sometimes need a bit of time to filter information, let people be aware of it, know that there’s work being done. But, it’s a bit like a drip feed process. But you don’t want that to be too long either, so finding the balance will be really challenging.* (Healthcare professional; 2030).

Participants indicated that the views of women and other stakeholders were critical to inform implementation. Obtaining acceptability was framed around controversies about relative harms and benefits; participants felt that introducing a low-risk pathway would likely regenerate much debate on the subject. This contention highlights the difficulties that those aiming to implement a low-risk pathway are likely to face.

*… we have to have everybody who’s anybody all singing from the same hymn sheet […*] *But we all know the sort of people who are anti-screening with the same sort of anti-screening message. Now, they’ll love it, but you’re going to get the pro screening lobby on the other hand who think this is a dreadfully daft idea.* (Healthcare professional; 2028).

#### Sub-theme 3.2: communication is essential

Given the expected challenges of implementing a low-risk pathway, communication was viewed by all as crucial, particularly to ensure public understanding. Most participants viewed this in light of how long breast screening has run as ‘one size fits all’; however, one participant felt that because risk is discussed in other programmes, such as antenatal screening, it will be straightforward to communicate risk-stratified screening to women.

*… it looks like when you start introducing risk based screening, there’s a whole new concept. I think a lot of the preparatory groundwork in terms of general principles of it is already out there.* (Academic; 2036).

Similarly, for women who receive a low-risk outcome, several participants felt they should be expected to have some responsibility for their breast health during extended intervals, as long as this was made clear.

*… there will be a challenge in educating the public, or women, about the risks of breast cancer, and the harms and benefits of screening. And making it very clear what the justification is, for why you’re wanting to increase the interval for women at low-risk.* (Academic; 2024).

The external influence of the media was viewed by all as playing a role in affecting how a low-risk pathway is perceived. Although risk-stratified screening could benefit many, it was felt that the media could overstate the impact of a single woman receiving an interval cancer diagnosis during extended screening. Similarly, all participants were concerned about how this could negatively impact programme credibility. It was therefore seen as important to involve the media during implementation preparation to ensure changes are appropriately portrayed.

*… we maybe don’t use the media enough because patients and women, the public get so much mixed, they get a lot of their information from the media and a lot of it is inaccurate or confused, or even the mainstream news channels, they distort things or abbreviate it*. (Healthcare professional; 2037).

#### Sub-theme 3.3: considering service implications

Participants readily described how risk-stratified screening for low-risk women could affect breast screening services, including staff, and often highlighted as already under pressure. This view was empahsised by participants with screening programme operational or management roles. All participants viewed increased screening intervals for low-risk women positively by reducing staff workload. However, when accounting for all risk groups, it likely has a neutral effect if services also screen groups at greater risk more frequently.

*… my sense of all of this is that what you’re doing is trying to increase the frequency for people, who are at higher risk and reduce it for people at lower risk […*] *I think probably in terms of screening visits, consultations and so on, the overall volume of work probably wouldn’t change all that much.* (Academic; 2023).

Additionally, many indicated that women may have risk-related questions at screening, for example risk changing over time, affecting appointment length. Overall, participants were less concerned about capacity for conversations about low-risk relative to other risk groups. Staff training would nonetheless be required to support conversations before, during and after screening appointments. A helpline or website were often cited as useful resources to mitigate impact on services given that face-to-face may be ideal but not practical for demanding services. By contrast, user involvement participants were skeptical that helplines would be useful. Primary care settings were also considered where GPs would need information on which pathway women were on to facilitate discussions; there was no consensus on the level of impact this would have.

*… you’re probably going to raise those questions, so you need to make sure that there are the resources and the capacity to have those conversations with women […*] *so that there is an opportunity for people, either, well, maybe it could be a telephone contact, or a face to face, to say, if you want to discuss it further, then you can either speak to somebody on the phone, or we can arrange for you to come and see somebody …* (Academic; 2024).

Should a risk-stratified approach be implemented, all participants discussed the need for monitoring procedures to ensure women are invited at the right time and allocated to correct pathways. This was always viewed as a serious risk given that current infrastructure was referred to as outdated; care should be taken to develop capable IT and administrative systems flexible enough to cope during delivery.

*They’ll need to get the letters right to whoever, you know? And not lose them out of the system. I don’t know, I mean, that’s the IT people and the programs, and how they write the program for … and how you input the data and god forbid there are slips, you know, that you plonk person X into that track.* (Screening operations/management; 2031).

## Discussion

This is the first in-depth exploration with national healthcare policy decision-makers around the feasibility of implementing risk-stratified screening for women at low-risk of breast cancer. The findings suggest that increasing the screening interval for women at low-risk is acceptable, in principle, from a wide range of professional perspectives. Participants did identify issues to be overcome prior to implementation, centred mainly on evidence required and service infrastructure capability. The entire sample recognised the importance of involving women who would be affected by such a change at the earliest opportunity.

Previous stakeholder workshops indicate that professionals seek evidence of the safety and cost-effectiveness of risk-stratified breast screening [19]. Current study findings highlighted expectations of high quality evidence demonstrating that extending screening interval for women at low-risk is safe to implement, with minimal risk of developing aggressive, less treatable cancers during longer intervals. Having this data could convince stakeholders that an extended interval for low-risk women is not underpinned by funding issues. Studies using interval cancers per stratified risk group as an outcome measure could determine whether a longer screening interval is not reducing the balance of benefits of screening for low-risk women in the absence of long-term follow up data required to report mortality. However, lead-time bias would affect the utility of this approach.

The concern that there is currently inadequate research demonstrating the accuracy and stability of models used to calculate breast cancer risk was also emphasised as a factor hindering implementation. This is understandable given that limitations regarding the accuracy and validity of risk models have recently been discussed [26–28]. However, on-going trials were highlighted as positive steps towards personalising breast cancer screening and a multi-country validation study found several breast cancer risk models could accurately predict breast cancer risk [29, 30]. National governments appear to be moving in this direction, as outlined in a recent UK consultation [31].

Other issues expressed by participants focused on having evidence of the number of interval cancers diagnosed during extended intervals and subsequent financial implications. Epidemiological evidence does suggest that women at low 10-year risk are less likely to be diagnosed with breast cancer compared to other risk groups [9] and modelling suggests it is likely to be cost-effective [15, 16]. It was difficult for participants to define a low-risk group. This is unsurprising given that different research groups lack consistency describing a low-risk cohort. For example, risk thresholds used vary [12, 32] and are calculated from different risk models, which are based on different risk factors [10, 33, 34].

Participants offered varied opinions on the potential consequences of increasing intervals for low-risk women. The main advantage would be to reduce unnecessary harms from breast cancer screening, both physical and psychological. However, this was often discussed in tandem with issues women and screening professionals may face if the screening interval was drastically extended. The potential negative impact of receiving breast cancer risk estimates on women’s worries, attitudes towards breast screening and subsequent attendance were highlighted as particular concerns. However, the available evidence suggests no adverse psychological harms when providing these at breast screening and the majority of women in a recent UK survey are interested in having their risk assessed [35, 36]. Decision-makers should ensure it is possible to effectively communicate that although women are at low-risk of developing breast cancer, they still have some risk. Women could be particularly negatively impacted if they are unclear that this is a real harm, potentially undermining screening overall, particularly if media sensationalise this. Concerns about receiving mixed messages from media and healthcare professionals has previously been identified by women considering risk-stratified breast screening [37]. However, some evidence suggests that women may be willing to have less frequent breast screening if found to be at lower risk via genetic testing [38, 39].

Other implementation-specific issues concentrated on current breast screening programme capacity and capability to deliver a service where women receive invites at different intervals. Previous reviews and workshops report potential implementation issues related to genetic-based risk-stratified breast screening highlighting similar organisational constraints [21, 40]. There was often a sense of conflict relating to how low-risk women should opt to have extended intervals grounded in what is ethical versus feasible to implement. A ‘proactive approach’ was previously regarded as most important to British breast screening professionals regarding women’s decision-making to participate in risk-stratified screening [18]. Involvement of women likely to be identified as low-risk in pathway development would ensure an acceptable proposal could be offered during implementation pilots.

### Strengths and limitations

Although international screening programmes were discussed, perspectives on implementation of risk-stratified screening are largely limited to a UK, mainly English context. Members of the research team (DGE, AH) have been involved in policy-level work regarding risk-stratified screening which may have influenced respondents’ interest in participating in the study and expressed opinions, although these team members were not involved in study data collection or analysis. Further, the team are involved in research in this area [41]. It could be that individuals who participated have a more favourable opinion of risk stratified breast screening, although the high rate of uptake of interviews by professionals with multiple demands on time suggests that this was not a major threat to the validity of these findings. Additionally, although multiple people working in primary care were approached to participate, the study sample does not constitute a representative perspective from this professional group. A concurrent study with similar research aims, conducted by the same research team, successfully recruited general practitioners from primary care to participate in focus groups.

These findings offer a specific perspective on the acceptability of extending breast screening intervals for low-risk women from key individuals who are involved in decision-making at a policy level. Other strengths include the variety of professional disciplines recruited to the study including two service user members of the UKNSC and the rigor employed to ensure representativeness of the sample involved in policy-level healthcare decision-making within the time constraints of the study. The open-ended interview approach allowed participants to express their opinions without being constrained, and the inductive analysis approach allowed these opinions to drive the analysis, rather than the opinions of the research team.

### Implications

Consensus approaches are required to outline a low-risk pathway defining which women would enter it before attempting roll out. High quality evidence demonstrating it is possible to accurately identify a low-risk group with no major adverse impact is required before a decision can be made about implementation. A barrier in producing such evidence is the limitation as to the design of such research studies; it will not be possible to conduct a randomised controlled trial where breast screening is withheld from some women. Extensive feasibility work to develop a low-risk pathway, multi-stakeholder communication strategies and evaluation frameworks are immediate priorities. Evidence is also required to establish whether and when risk should be re-assessed. Consultations with women to specifically focus on low-risk rather than risk-stratified screening more generally will gauge acceptability and ensure equality of access.

## Conclusions

The present study has identified a number of uncertainties that need to be resolved before implementation can take place. These centred on demonstrating accurate identification of low-risk women, gaining acceptability from women, evidencing of lack of harm and ensuring current breast screening programmes have the capability to cope with women on different screening length intervals depending on risk.

## Supplementary information

**Additional file 1.**

## Data Availability

Requests may be made from reputable researchers with justification to access the anonymised dataset for secondary analysis. Requests should be made to the corresponding author in the first instance.

## Abbreviations

GP: General Practitioner
NHSBSP: National Health Service Breast Screening Programme
NICE: National Institute for Health and Care Excellence
TC: Tyrer-Cuzick
UKNSC: UK National Screening Committee
