# Supplementary material for: Risk stratified breast cancer screening: UK healthcare policy decision-making stakeholders’ views on a low-risk breast screening pathway
Source: BMC Cancer. 2020 Jul 22;20:680. doi: 10.1186/s12885-020-07158-9 (PMC7374862; doi:10.1186/s12885-020-07158-9)
Supplement: Supplementary file 1 — Additional file 1. [file 12885_2020_7158_MOESM1_ESM.docx]

**Additional file 1 Interview topic guide**

**Views on the appropriateness and safety of recommending an increased screening interval to low risk women in the NHS breast screening programme.**

**Introduction to topic**

Thank you for agreeing to meet me today. As you may know we are currently running a study where across 5 sites in the North West of England women are given the opportunity to find out their risk of developing breast cancer. We are able to calculate that by obtaining information on lifestyle and family history and combining that with women’s mammographic breast density. Providing women with their risk is especially important for high risk women as they could have access to increased screening, of once every 12/18 months, or they could be offered chemoprevention medication.

It has been suggested that reducing the frequency of screening that low risk women receive could be beneficial to women. And as there appear to be more harms associated with breast screening that benefits in the form or false positives and unnecessary treatments, an increased screening interval that goes beyond the current 3 year guideline could actually maximise benefits and minimise these harms.

This study is running alongside the study I have outlined. The aim of today therefore is to find out from yourself what you think about this proposal and what would be acceptable in your eyes. However we are interested in your thoughts and opinions and what you would deem acceptable. We are also interested in how this proposal would be communicated to women and what information they would need to make a decision.

I also have to mention that in the unlikely event that anything is discussed that constitutes mal-practice or misconduct I have a duty to report such a disclosure and would therefore have to break confidentiality.

Do you have any questions before we begin?

**Discussion points**

**Introduction to topic**

- What is the feasibility of introducing an increased screening interval beyond 3 years for low risk women into the NHSBSP?

**Implementation criteria**

- If this were to be implemented, what criteria should inform this change? What should we consider? (**prompts:** Financial cost of screening; Number of missed/interval cancers; Number of overdiagnosed; mortality)

Risk Threshold

- What risk threshold should be used to recommend an increased screening interval?
- Low risk women could be at different ends of the categories, i.e. some women could be at 0.5% risk of breast cancer and others at 1.5%. What do you think about this? (**Prompt:** consideration when suggesting less frequent screening?)

Interval length

- What would be an acceptable length between screening intervals?
  - When should it be introduced? (**prompt:** age of 1st screen)
  - When should risk be re-evaluated?

Implementation

- How do you think a screening programme like this should be implemented? / How do you see this working within the NHS BSP?

Information provision

- What information do you think women would need in order to make a decision about making a change in their screening? **/** What should women know about why an increased screening interval is being recommended? (**prompt**: cost of high risk screening; ‘safety’ /Harms of screening)
- How should women receive information about this proposal? **Prompts:**
  - Via a letter or leaflet?
  - Face to face?

Informed decision-making

- What is the best way to facilitate informed choice? (**prompt:** own decision; HCP advice; PHE advice; both)
- How can women be best supported, especially women from BAMER backgrounds and women of low SES.
- What advice should healthcare professionals give?
- How should women opt for increasing their interval between screenings? (**prompt:** Opt in or opt out)
  - Should this be open to change?
  - How could women be able to change it?

**Implications of risk-stratified screening**

- What are the benefits to low risk women if they were to opt for an increased screening interval?
- What service implications do you foresee with this proposal? (**prompt:** capacity/workload; financial’ high risk women; less overdiagnosis?)
- What issues could women face (and how could they be mitigated)?
- What issues could healthcare professionals encounter (and how could they be mitigated)?
- What issues could policy makers encounter (and how could they be mitigated)? (**Prompt:** media, lobby groups, trust, credibility)
- What do you think is the most important consideration for implementing risk stratified screening? (**prompt:** screening interval; risk threshold; age at which women start)

***Note- always explore natural areas of important discussion.***

**Closing comments**

Thank you again for meeting me today and sharing your views. Your insights and discussions will be invaluable when we could to pilot this initiative.

- Was there anything that you would like to add? Any questions?
- Was there anything that you thought we would discuss and haven’t?
